# Biomarker-driven feedback control of synthetic biology systems for next-generation personalized medicine

**DOI:** 10.3389/fbioe.2022.986210

**Published:** 2022-09-26

**Authors:** Bozhidar-Adrian Stefanov, Martin Fussenegger

**Affiliations:** ^1^ ETH Zürich, Department of Biosystems Science and Engineering, Basel, Switzerland; ^2^ Faculty of Life Science, University of Basel, Basel, Switzerland

**Keywords:** synthetic biology, gene switches, gene regulation and expression, cell-based therapies, personalised medicine

## Abstract

Many current clinical therapies for chronic diseases involve administration of drugs using dosage and bioavailability parameters estimated for a generalized population. This standard approach carries the risk of under dosing, which may result in ineffective treatment, or overdosing, which may cause undesirable side effects. Consequently, maintaining a drug concentration in the therapeutic window often requires frequent monitoring, adversely affecting the patient’s quality of life. In contrast, endogenous biosystems have evolved finely tuned feedback control loops that govern the physiological functions of the body based on multiple input parameters. To provide personalized treatment for chronic diseases, therefore, we require synthetic systems that can similarly generate a calibrated therapeutic response. Such engineered autonomous closed-loop devices should incorporate a sensor that actively tracks and evaluates the disease severity based on one or more biomarkers, as well as components that utilize these molecular inputs to bio compute and deliver the appropriate level of therapeutic output. Here, we review recent advances in applications of the closed-loop design principle in biomedical implants for treating severe and chronic diseases, highlighting translational studies of cellular therapies. We describe the engineering principles and components of closed-loop therapeutic devices, and discuss their potential to become a key pillar of personalized medicine.

## Introduction

The development of polypeptide-based therapeutics such as antibodies and hormones has opened up exciting avenues for treating diseases previously considered incurable (Fosgerau and Hoffmann, 2015; Carter and Lazar, 2018). However, the greatest limitation for such therapies often lies in the use of traditional delivery and control systems, which may either generate undesirable spikes in drug concentration or deliver a delayed response (Ryman and Meibohm, 2017). In marked contrast, most physiological processes are tightly regulated by control circuits that employ positive or negative feedback based on a physiological parameter, such as glucose concentration (Saltiel and Kahn, 2001) or body temperature (Stefanov et al., 2021) to regulate a physiological response, such as insulin secretion (Saltiel and Kahn, 2001) or heat dissipation (Stefanov et al., 2021). Such control circuits generally offer excellent response kinetics and tight control of the concentration of the released physiological effectors (El-Samad, 2021). These features have stimulated synthetic biologists to engineer systems that reroute natural mechanisms of response-loop regulation to deliver therapeutic agents on demand (Xie and Fussenegger, 2018). For example, specialized receptors that respond to disease markers can be rerouted into downstream cellular effectors for the feedback-adjustable release of a therapeutic agent (Kemmer et al., 2010; Kitada et al., 2018).

**FIGURE 1:**
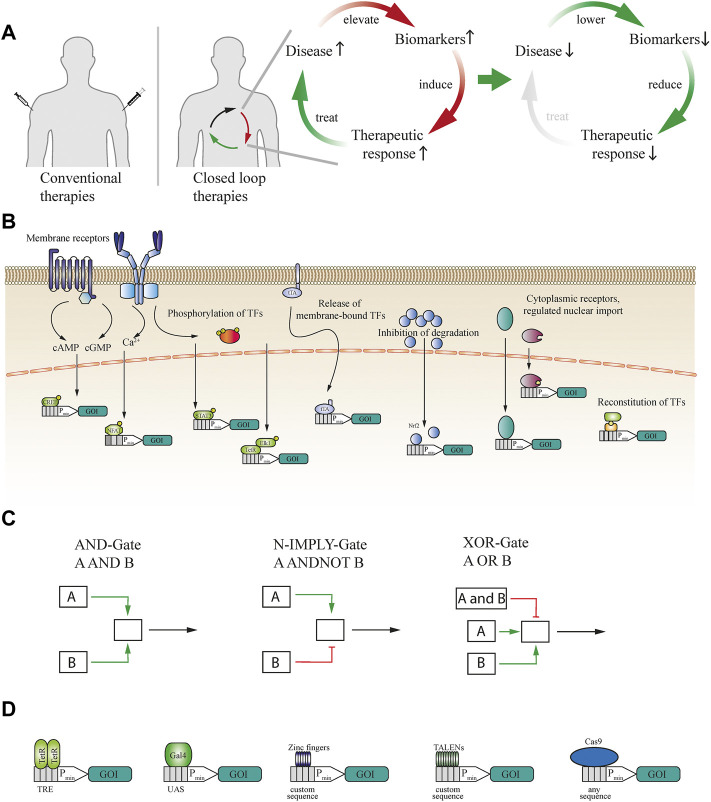
**(A)** Schematic illustration of conventional therapeutics versus closed-loop-operated therapeutics. While standard therapies often rely on invasive injections and estimated dosages, closed-loop cellular therapies can enable non-invasive and precise dosing of the therapeutic proteins. Elevated disease biomarkers induce the therapeutic response, which then ameliorates the disease condition, self-limiting the therapeutic response and closing the circuit. **(B)** Sensing of biomarkers can be achieved through multiple modes and cellular receptors. Extracellular antigens can be sensed either by natural receptors such as GPCRs or, if none exist, by engineered generalizable receptors. These receptors activate second messenger pathways, such as those mediated by cAMP, cGMP or calcium, or induce the post-translational modification of downstream effectors. Intracellularly available biomarkers can induce the release of transcription factors (TFs) from the cellular membrane, inhibit the degradation of TFs, activate or induce transport into the nucleus, or reconstitute split TFs. **(C)** Signalling from activated receptors can also be rerouted into logic gates to enable multilayered operations. For example, AND gate logic requires simultaneous activation of two different receptors or TFs. In the case of N-IMPLY gate logic, a receptor induces the system only if another receptor is not active or present. XOR gate logic takes input from two receptors, either of which can separately activate the system, whereas both simultaneously cannot. **(D)** To expand the scope of synthetic biology systems, the DNA-binding domain (DBD) can be exchanged. TetR DBD can be used to trigger gene expression from a TRE-flanked minimal promoter, to which it binds as a dimer. A monomeric DBD such as Gal4 can be used to control gene expression from a UAS-flanked minimal promoter. Various zinc finger and TALEN proteins can be used to bind custom DNA sequences. To expand the range of binding sites even further, any DNA sequence can be bound by dCas9 if a suitable guide RNA can be designed.

Thus, closed-loop designs, as schematically illustrated in Figure 1A, are considered to have enormous potential (Sedlmayer et al., 2018a). Indeed, closed-loop feedback has already been established as beneficial even for conventional therapies based on small-molecule drugs. For example, in a comparative study, the adjustment of administered drug amounts on the basis of laboratory measurements significantly increased the reduction of viral load in AIDS patients (Caetano and Yoneyama, 1999). However, the use of closed-loop control in conventional therapy requires frequent and labor-intensive laboratory testing. This has led to increased interest in mixed approaches, in which the release of therapeutic cargo from hydrogels (Ehrbar et al., 2008) or cellulose capsules (Fluri et al., 2008) is coupled to a biomarker-triggered activation process. The activated domain interferes with the hydrogel’s stability or induces a cellulase for capsule degradation to cause release of the cargo in an amount proportional to the degree of activation. For this purpose, a connection between a biomarker sensor, such as a synthetic receptor, and a biological effector has to be established, by using either a bioprocessable container, such as liposomes (Saad et al., 2008), or a mechanical or non-cellular “biocomputer,” such as electronic devices utilizing immobilized enzymes (Kim et al., 2018), mesoporous silica nanoparticles (Zhao et al., 2009; Cheng et al., 2015), glucose-responsive polymersomes (Kim et al., 2012) or polymeric PEG hydrogels activated by thrombin (Maitz et al., 2013), or lipase (Yu et al., 2018) to enable closed-loop control based on the enzymatic activity in serum. Enzymatic reactions can also be used with pH- or urea-responsive biopolymers (Zhao and Li, 2008). Alternatively, hydrogels can be functionalized with, or constituted from, special amino acids that swell the gel in response to decreasing pH and thereby increase the release of the entrapped substance (Rizwan et al., 2017). Sensing the pH might be useful for targeted delivery to body areas of specific pH, such as the intestine, or in the case of diabetic ketoacidosis. However, a major drawback of these mixed approaches is that they are not self-replenishable and cannot function as prosthetic implants for the long-term treatment of chronic disease.

In contrast, cell-based therapies are not only capable of continuous *in-situ* monitoring of biologically relevant markers, but also are self-sustainable once implanted (Kojima et al., 2020), allowing for prolonged autonomous function. In addition, polymodal effector actions can be employed, such as the release of therapeutics combined with paracrine cell-cell communication (Kojima and Fussenegger, 2019). Some of the most innovative clinical breakthroughs involve feedback loops integrated into cells, as exemplified by the successful application of the closed-loop regulation principle in CAR-T cell therapy by introducing a cascade, such that antigen detection on cells in a certain environment results in the detected cells being killed. The effector cells are not consumed in the process, but continue screening for other cells that express the antigen (Jackson et al., 2016; June et al., 2018). This is superior to an alternative approach, in which viruses recognizing specific combinations of cell-surface markers were used to activate cytolytic activity, but were consumed in the process (Hiss and Fielding, 2012).

In this review, we outline the design features and components required for closed-loop control in living cells and summarize recent advances in the construction of such systems, focusing on those that have been successfully tested in animal models. We also discuss the prospects for such systems in next-generation personalized medicine.

## Design principles of cellular closed-loop therapeutics

Three main categories of components - sensor, processor, and effector - are required for cellular closed-loop therapeutics. In the first category, sensory components include endogenous or artificially engineered receptors that interact with and facilitate the detection of biomarkers and convert biomarker concentration into a cellular signal, which may be the induction of second messengers such as cAMP, cGMP, or Ca^2+^ (Rössger et al., 2013a; Kim et al., 2015; Xie et al., 2016), activatory or inhibitory phosphorylation of downstream proteins (Chassin et al., 2017; Scheller et al., 2018), import of transcription factors into the nucleus (Theodore et al., 2008), release of membrane-bound transcription factors (Li et al., 2013), inhibition or induction of protein degradation (Gao et al., 2018) or reconstitution of transcription factors (Donahue et al., 2020) (Figure 1B). In addition, because microorganisms such as pathogenic bacteria have evolved the ability to sense various aspects of their host environment (Balasubramanian et al., 2017), these sensors can be repurposed to detect small molecules such as antibiotics (Weber et al., 2005), bile acids (Rössger et al., 2014) or amino acids (Hartenbach et al., 2007) that are linked to the host’s metabolism. An important recent advance in engineered sensors was the development of generalizable extracellular receptors, in which specific single-chain variable fragments (scvf) are fused to the extracellular domain of an erythropoietin-insensitive erythropoietin receptor scaffold, enabling various proteins, such as the cancer biomarker prostate-specific antigen (PSA), and small molecules to activate intracellular signaling pathways in mammalian cells (Scheller et al., 2018). Furthermore, the intracellular signaling domain can be exchanged for others from human cytokine signaling receptors to provide signal amplification and increase the sensitivity of the receptors.

In the second category, the role of processing components in closed-loop systems is to reroute sensor activation by molecular cues to gene expression or activation of effector components. These regulatory networks are required not only to integrate the biomarker cues provided by the sensor in order to activate signal transduction and transcription initiation, but also to deactivate the system upon recovery from the pathological condition, allowing the therapeutic cells to return into their inactive equilibrium state. Although challenging, engineering of such gene circuits in mammalian cells has seen remarkable progress in recent years (Ausländer and Fussenegger, 2016). Logical computations in mammalian cells using various logic gates including AND, N-IMPLY, and XOR have been described and can enable programming of complex circuits to control cellular therapies (Ausländer et al., 2012) (Figure 1C). A significant advantage of logic gates is the increased specificity since multiple conditions have to be fulfilled in a specific way for the downstream activation. Such circuits initiate gene expression by employing transactivation factors fused to DNA-binding domains that recognize binding sites upstream of inducible promoters. Both bacterial (TetR) and yeast (Gal4) DNA-binding domains are widely used (Kakidani and Ptashne, 1988; Ramos et al., 2005). Recently, DNA-binding domains based on TALENs (Moore et al., 2014) and zinc fingers (Israni et al., 2021) have also attracted attention, as they can bind to customizable sequences and show low immunogenicity (Figure 1D). Alternatively, rerouting of intracellular signaling cascades into chimeric promoters with binding sites for endogenous transcription factors for the expression of transgenes has been used when the sensory components are endogenous hormone or cytokine receptors (Saxena et al., 2016; Scheller et al., 2018). Intrinsic pathways can also be rewired *via* engineered fusion complexes based on dCas9 and an MS2 aptamer-modified guide RNA that is recognized by a transactivator-fused MS2 coat protein (MCP) to trigger gene expression from various endogenous promoters and permit therapeutic cell reprogramming (Krawczyk et al., 2020a) (Figure 1D). These developments have enabled the establishment of precise gene circuits that can integrate biomarker-induced signaling into engineered cells and translate it to either orthogonal or endogenous gene expression.

The third category, the effector components, are the therapeutic outputs, whose precise delivery timing and dosage determine the success of the therapy (Kojima et al., 2020). Effector proteins already used in cellular therapies include antibodies (Saxena et al., 2016; Carter and Lazar, 2018), nanobodies (Xenaki et al., 2021), hormones (Stefanov et al., 2021), antimicrobials (Liu et al., 2018a), and other functional peptides (Lau and Dunn, 2018; Ye et al., 2013). Since many of these, especially antibodies, are complicated and expensive to produce and purify, cellular therapies may use cell types optimized for this purpose. In addition, the effector can take the form of direct activation of cellular functions, such as cancer cell-killing in CAR-T therapy (Jackson et al., 2016).

Overall, synthetic gene circuits provide almost limitless opportunities to design and engineer combinations of sensor, processor, and effector components for cellular closed-loop therapeutics, including complex intracellular biocomputing to take account of either the presence or absence of multiple inputs (Manhas et al., 2022). To illustrate how the closed-loop design principles are implemented in real-life applications, we will next summarize recent developments in the field. Some of the most prominent translational work on closed-loop cellular therapeutics, covering a wide range of diseases, is listed in Table 1.

**TABLE 1:** Overview of closed-loop-compatible cell therapies with potential for translation to treat complex diseases.

Condition and biomarker	Molecular mechanism of the closed-loop system	Reference
Gout, urate	Uric acid levels were detected by a modified KRAB-HucR repressor from *D. radiodurans*. Urate-dose-dependent induction of a secretion-engineered *A. flavus* urate oxidase enabled the removal of uric acid in hyperuricemic mice to afford stable sub-pathologic levels, resulting in a decrease of uric acid crystal deposits in the kidneys.	Kemmer et al. (2010)
Ovulation, luteinizing hormone	Luteinizing hormone receptor signaling was rerouted to CREB1-controlled expression of cellulase for the *in utero* release of cellulose-encapsulated spermatozoa, enabling ovulation-inducible fertilization in Swiss dairy cows.	Kemmer et al. (2011)
Diet-induced obesity, fatty acids	An intracellular lipid sensing receptor was generated by fusion of PPARα and the phloretin-responsive repressor TtgR. Pramlintide was reversibly expressed for appetite reduction from a TtgR-controlled minimal promoter, and gene expression was induced by PPARα depending on the fatty acid concentration.	Rössger et al. (2013a)
Diabetes associated ketoacidosis, pH	Signaling from the proton-activated cell-surface receptor TDAG8 was rewired into a cAMP-sensitive response element operated by CREB1 for the expression of transgenes, including insulin for the treatment of diabetes-associated ketoacidosis.	Ausländer et al. (2014a)
Allergy, histamine	Histamine H2 receptor (HRH2)-based detection of histamine released from immune cells upon allergic IgE activation of basophils rewired into a cAMP-sensitive minimal promoter for the expression of transgenes.	Ausländer et al. (2014b)
Psoriasis, TNF, and IL22	Sensing of TNFα and IL22 serum levels using the corresponding receptors and linking their downstream signaling in an AND-gate logic enabled the expression of the anti-inflammatory cytokines IL4 and IL10 to treat psoriasis or prevent its development.	Schukur et al. (2015)
Topical ointments application, parabens	Paraben-inducible repressor PmeR from *P. syringae* pathovar tomato DC3000 and its cognate operator OPmeR were used to enable transgene expression in mammalian cells in response to topical ointments containing parabens. These cells may be programmed to enable a closed-loop behavioral response to topical ointments.	Wang et al. (2015)
T1D and T2D, glucose	Ectopic expression of the calcium voltage-sensitive channel Cav1.3 in HEK-293 cells proved sufficient to enable glucose-dependent depolarization, triggering intracellular calcium signaling. This was rerouted to NFAT-operated minimal promoters for the expression of GLP-1 or mouse insulin to treat the hyperglycemia-inducing condition.	Xie et al. (2016)
Liver damage, bile acids	Bile acid-inducible signaling from G protein-coupled bile acid receptor 1 (TGR5) was rewired into an engineered promoter controlling transcription of the hepatocyte growth factor (HGF) to foster repair of damaged tissue.	Bai et al. (2016)
Graves’ disease, T3	The system consists of a synthetic thyroid-sensing receptor (TSR), a fusion protein based on Gal4 DNA binding domain and the human thyroid receptor-α. This sensor can monitor T3 and T4 serum levels, and it induces the expression of a thyroid-stimulating hormone receptor antagonist (TSHAntag) to inhibit thyroid-stimulating hormone or human autoantibody-mediated activation of TSHR, thus reducing T3 and T4 serum levels.	Saxena et al. (2016)
Insulin resistance, insulin	Detection of pathological insulin concentrations by the insulin receptor enabled activation of the MAPK signalling pathway and a chimeric TetR-Elk1 fusion transcription factor that induces the expression of an insulin signaling-sensitizing hormone, adiponectin, from a TetR-operated minimal promoter.	Ye et al. (2017)
Arthritis, TNF	Stem cells were engineered to sense and antagonize TNFα-mediated joint inflammation through closed-loop control using TNFα receptor-triggered NF-kB signaling. A soluble signal-transduction-deficient TNFα receptor was CRISPR-integrated into the NF-kB-inducible ccl2 promoter, which is activated by TNFα signaling.	Brunger et al. (2017)
Allergy, IL-4, and IL-13	The system monitored extracellular levels of the IL-4 and IL-13 pro-inflammatory cytokines and upon activation by pathophysiological concentrations of these cytokines, the engineered cells responded with the secretion of DARPin E2_79, a protein that binds human IgE and antagonizes histamine released from basophile granulocytes.	Chassin et al. (2017)
Senescent phenotypes, IL-6	A chimeric IL-6 receptor fused to the intracellular signaling domain from VEGFR2 was used to generate a Ca^2+^ signal in response to IL6 stimulation. Co-expression with an engineered Ca^2+^-activated RhoA enabled actin remodeling and directed migration towards the source of IL-6.	Qudrat et al. (2017)
Cancer, HER2 antigen	Nonimmune cells were engineered with a HER2 receptor for IL-4/IL-13R-induced STAT6 signaling, inducing the expression of VP22-FCU1 cytotoxic effector protein to eliminate the detected HER2-expressing cells.	Kojima et al. (2018)
Pathogenic biofilms, N-formyl peptides, and PAI-1	Mammalian cells were used to detect formyl peptides and program interference with quorum-sensing of *V. harveyi* and to prevent biofilm formation by the pathogenic yeast *C. albicans* through release of autoinducer 2 (AI-2)*.*	Sedlmayer et al. (2018a)
	To program dysregulation of quorum sensing by *P. aeruginosa,* its PAI-1 was detected by the *Pseudomonas* autoinducer sensor PAiS expressed ectopically in engineered mammalian cells and rewired into LasR-operated promoters for the expression of an autoinducer-inactivating enzyme (MomL) and the biofilm-degrading hydrolases PslG and PelA.	Sedlmayer et al. (2017)
MRSA infection, cell wall parts	Rewiring of human Toll-like receptors TLR2, TLR1, TLR6 signaling enabled the detection of gram-positive bacterial wall components present on MRSA bacteria, and the rerouting of downstream NF-kB and AP-1 signaling into an inducible promoter for adjustable expression of the bacteriolytic enzyme lysostaphin.	Liu et al. (2018a)
Hyperlipidemia, fatty acids	A free fatty acid-sensitive receptor (GPR40) triggers intracellular calcium signaling that activates trangene expression from an NFAT-controlled minimal promoter.	Liu et al. (2018b)
Fever, Increased temperature	An engineered TlpA protein sensor enabled the detection of temperatures in the physiological fever range by utilizing the temperature-reversible inhibition of the fused tTA complex (TetR transactivator) for generalizable transgene expression from TetR-controlled promoters.	Stefanov et al. (2021)

AI-2, autoinducer-2; AP-1, activator protein 1; cAMP, cyclic adenosine monophosphate; Cav1.3, calcium channel, voltage-dependent, L type; CD14, cluster of differentiation 14; CREB1, cAMP-responsive element binding protein 1; CRISPR, clustered regularly interspaced short palindromic repeats; DARPin, designed ankyrin repeat protein; Elk1, ETS like 1; GLP-1, glucagon like peptide 1; GPR40, free fatty acid receptor 1; HER2, human epidermal growth factor receptor 2; HGF, hepatocyte growth factor; HRH2, histamine receptor H2; HucR, *D. radiodurans* urate-sensing repressor; IgE, immunoglobulin E; IL-1, interleukin-1; IL-10, interleukin-10; IL13R, interleukin-13R; IL-22, interleukin-22; IL-4, interleukin-4; IL-, interleukin-6; KRAB, Krüppel-associated box; MAPK, mitogen-activated protein kinase; MRSA, methicillin-resistant *Staphylococcus aureus*; NF-kB, nuclear factor kappa B; NFAT, nuclear factor of activated T cells; PAI-I, *P. aeruginosa* autoinducer 1; PAiS, *Pseudomonas* autoinducer sensor; PPARα, peroxisome proliferator-activated receptor-α; RhoA, ras homolog family member A; STAT6, signal transducer and activator of transcription 6; T3, triiodothyronine; T4, thyroxine; TDAG8, T cell-death-associated gene 8; TGR5, G-protein-coupled bile acid receptor; TLR1, toll-like receptor 1; TLR2, toll like receptor 2; TLR6, toll-like receptor 6; TNFα, tumor necrosis factor; TSHR, thyroid-stimulating hormone receptor; TSR, thyroid-sensing receptor; TtgR,l phloretin-responsive HTH-type transcriptional regulator; VEGFR, vascular endothelial growth factor receptor; VP22-FCU1, virus protein 22-fusion suicide gene.

### Closed-loop control of metabolic disease

**FIGURE 2:**
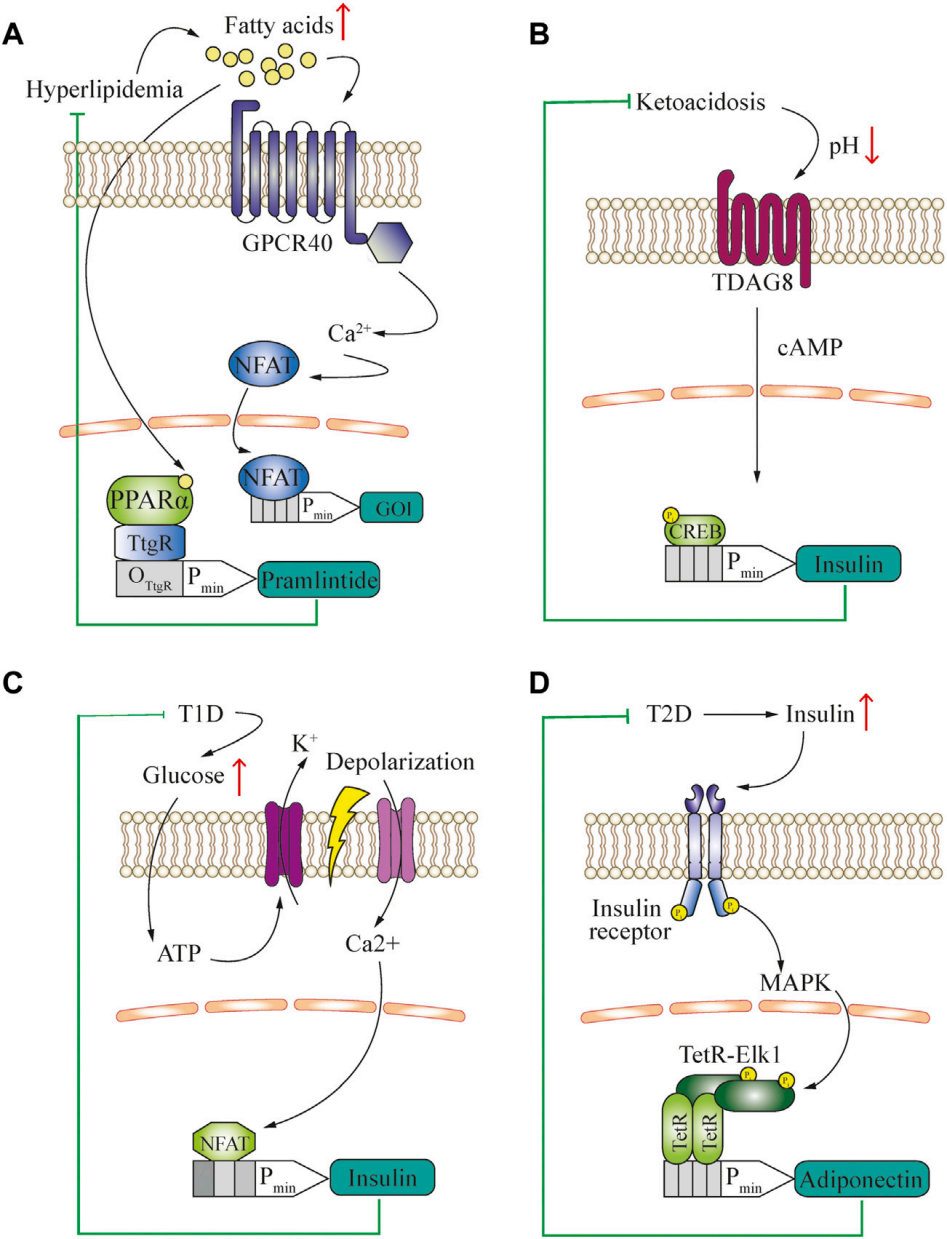
**(A)** Engineered closed-loop approach for the treatment of hyperlipidemia. The characteristic increase of blood free fatty acid concentration is used as a biomarker to activate GPCR40 and reroute the subsequent calcium signaling to an NFAT-operated promoter for the expression of a gene of interest (GOI) to deliver a therapeutic protein. Alternatively, a fusion transcription factor of TtgR DNA-binding domain and PPARα-sensing domain is used to control the expression of an appetite-reducing drug, pramlintide. **(B)** Engineered closed-loop approach for the treatment of type-1 diabetes-associated ketoacidosis. The characteristic decrease of blood pH is used as a biomarker. Through activation of TDAG8 in engineered cells and rerouting of cAMP signaling for insulin production, a closed-loop ketoacidosis response loop was established. **(C)** Engineered closed-loop approach for the treatment of type-1 diabetes. The characteristic increase of glucose concentration is used to generate a closed-loop circuit for the release of insulin. The hyperglycemia-induced intracellular increase in ATP triggers potassium efflux through ATP-gated channels, resulting in cell depolarization. This causes opening of voltage-gated calcium channels and induction of an NFAT-operated promoter, leading to the release of insulin. **(D)** Engineered closed-loop approaches for the treatment of type-2 diabetes. The characteristic increase of blood insulin concentration is used as a biomarker and activates insulin receptor signaling in engineered cells. This involves activation of the MAPK pathway, and subsequent activation of a chimeric TF consisting of Elk1 fused to the TetR DNA-binding domain, which drives the expression of adiponectin.

Metabolite-controlled gene networks have the potential to complement and interact with endogenous processes in patients. In this context, the concentration of free fatty acids (FFA) in the blood is an important biomarker of nutritional status, and has various potential applications (Figure 2A). For example, human GPCR40 can convert physiologically relevant changes in FFA concentrations to intracellular calcium signaling in mammalian cells (Liu et al., 2018b). This second messenger can be rerouted to induce the expression of a therapeutic protein that counteracts the increase in FFA. In an alternative approach, the peroxisome proliferator-activated receptor-α (PPARα) was fused as a lipid-sensing domain to the TtgR DNA-binding domain and this fusion induced gene expression of the appetite-reducing peptide-hormone pramlintide in diet-induced obese mice, reducing their food consumption and serum lipid concentrations (Rössger et al., 2013b) (Figure 2A). Obesity, although not usually considered a disease in itself, is a major risk factor for the development of metabolic, cardiovascular and autoimmune disorders (Grundy, 2004; Versini et al., 2014). Therefore, engineered cell therapy that protects against diet-induced obesity could provide a novel approach for treating metabolic syndrome. In addition, sensing of bile acids, another metabolic marker, was rerouted into different gene expression patterns by using *Campylobacter jejuni*-derived bile acid sensors (Rössger et al., 2014).

Another approach to sense the metabolic state of an organism is based on the measurement of physiological parameters such as pH and CO_2_ concentration in the blood, because these parameters reflect energy utilization. For example, diabetes can cause ketoacidosis, which can be used as a biomarker detectable by a pH sensor and rerouted to a chimeric promoter to produce and deliver a therapeutic polypeptide such as GLP-1 or insulin. A practical method to achieve this within the physiological range was developed by rerouting the human proton-activated cell surface receptor TDAG8 to a CREB1-operated chimeric promoter (Ausländer et al., 2014a) (Figure 2B).

In addition to slowing down the development of metabolic disease, closed-loop prosthetic implants have the potential to substitute for the functions of damaged cell types, such as pancreatic *β*-Cells in type-1 diabetes. To successfully treat this condition, the engineered cells need to release insulin upon detecting increased blood glucose. In a ground-breaking translational study, the increased ATP concentration resulting from high levels of intracellular glycolysis was used to activate ATP-gated K^+^ channels, which initiated depolarization of the cells. This depolarization was coupled to calcium signaling *via* ectopic expression of the voltage-gated calcium channel Ca_V_1.3. The increase in intracellular calcium was then rerouted to a chimeric promoter operated by NFAT for the expression of insulin, which was able to restore normal insulin levels in the blood and thus effectively treat type-1 diabetes (Xie et al., 2016) (Figure 2C). In contrast, an excessive insulin concentration in the blood is a hallmark of type-2 diabetes, and insulin-insensitivity caused by hyperinsulinemia is observed even in the early stages of the disease. Thus, hyperinsulinemia can be used as a biomarker to drive cellular therapies that target and prevent the progression of this condition. Ye et al. (2017) coupled insulin receptor signaling with the downstream MAPK-mediated activation of a fusion protein consisting of the transcriptional activation domain (TAD) of the Elk1 transcription factor and the TetR DNA-binding domain to induce the expression of adiponectin from designer cell implants, which ameliorated the development of insulin resistance (Figure 2D).

### Closed-loop control of the immune system

**FIGURE 3:**
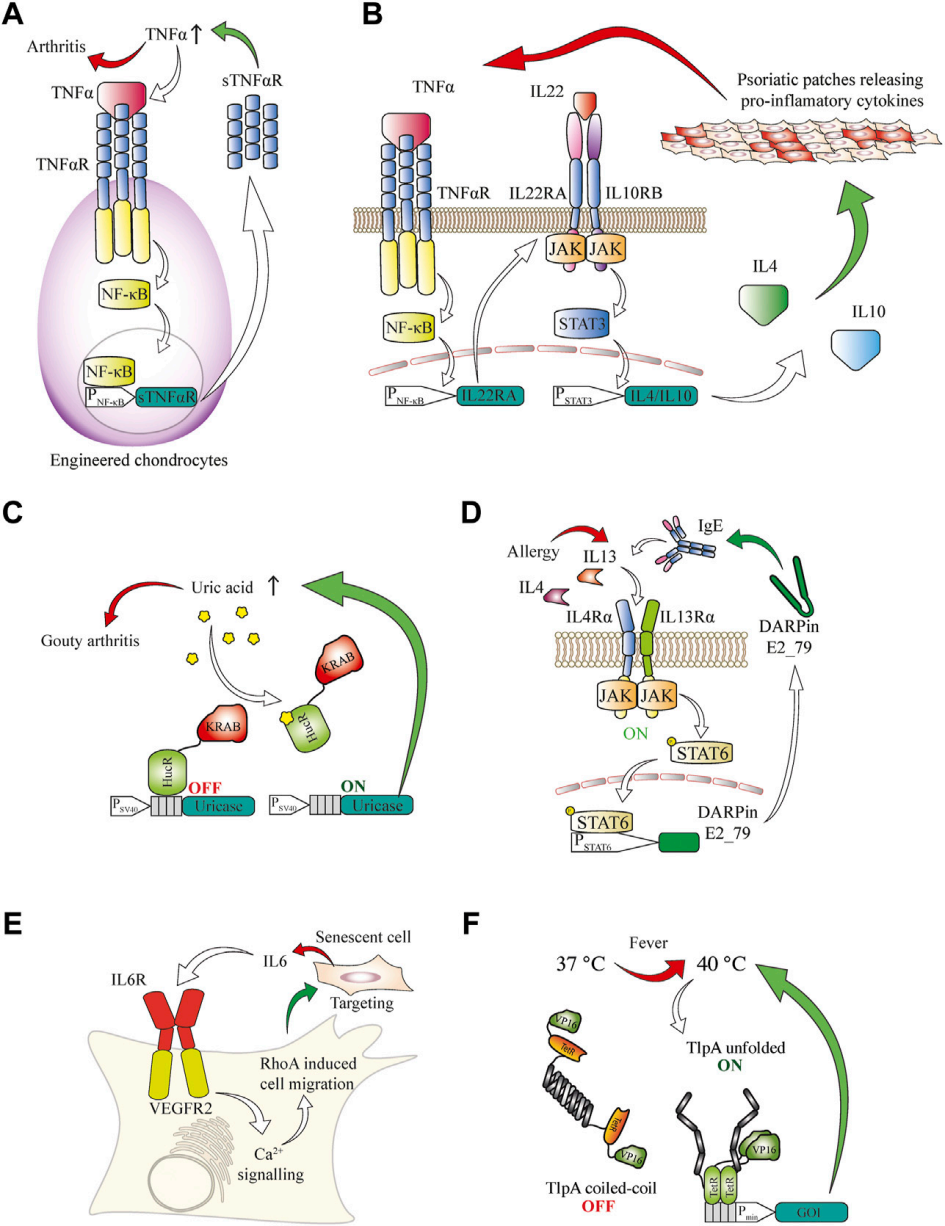
**(A)** Engineered chondrocytes generate an anti-inflammatory response in arthritis. Localized joint inflammation and the release of TNFα are among the main drivers for the development of arthritis. Chondrocytes are engineered to express TNFαR, and its signaling is routed *via* the NF-κB pathway to a chimeric promoter that induces expression of an inactive soluble TNFαR. This competes with active TNFαR for TNFα binding, thereby counteracting the inflammation in a closed control loop. **(B)** AND gate logic-based closed-loop cellular treatment for psoriatic skin patches based on the TNFα and IL22 cytokines. Increased TNFα concentration in the blood induces the expression of the IL22RA receptor subunit in engineered cells. This enables the sensing of IL22, which, together with TNFα, is a signature cytokine in psoriasis. Upon activation of this receptor the induced STAT3 signaling is rerouted to a chimeric STAT3 promoter for the expression of the anti-inflammatory cytokines IL4 and IL10, which suppress psoriatic flares. **(C)** Closed-loop cellular therapeutic circuit to counteract increased blood concentration of urate associated with gout. A fusion protein of the HucR urate-sensitive repressor from *D. radiodurans* with the KRAB inhibitory domain is bound to the HucR operator after an SV40 promoter and represses transcription under normal conditions. In the presence of inducing urate concentrations, the repressor is released from the DNA and *A. flavus* urate oxidase is produced and secreted, effectively reducing the urate concentration, which leads to repression of the promoter. **(D)** Closed-loop anti-allergic response by engineered mammalian cells. The presence of allergy-associated cytokines such as IL4 and IL13 can be sensed through the type II IL4R, which in turn activates the STAT6 signal transduction cascade. A minimal promoter operated by STAT6 induces expression of IgE targeting DARPin E2_79 to prevent further degranulation of immune cells and escalation of the allergic response. **(E)** Targeting of senescence phenotypes. Sensing of senescence-associated cytokines such as IL6 is enabled in engineered cells expressing a fusion receptor consisting of the IL6 extracellular domain and the VEGFR2 intracellular signaling domain. Upon activation, the receptor triggers calcium signaling and enables cellular migration towards the senescent cells through a modified calcium-sensitive RhoA for cytoskeletal remodeling. **(F)** A fever-inducible response is enabled through a genetically encoded thermometer to deliver closed-loop cellular therapies. The temperature-sensing transcription factor consists of tTA transactivator DNA-binding domain fused to an TlpA coiled-coil temperature sensor domain, which progressively unfolds at temperatures higher than 37° and enables transcription from a TetR-operated promoter.

Small molecules play important roles in many signaling pathways of various organisms and can be integrated into closed-loop circuits in engineered mammalian cells. For example, parabens are small-molecule preservatives frequently used in cosmetics and topical pharmaceutical products that are used to treat immune-associated irritated skin conditions. Such compounds can be sensed by designer cells equipped with bacterial sensors that detect plant defense molecules of the paraben class and the sensor signaling can be rerouted to deliver a therapeutic protein (Wang et al., 2015). Immunologically active small-molecule messengers such as histamine are another class of biomarker molecules that are readily detectable by protein sensors in engineered mammalian cells (Ausländer et al., 2014b). Detection of immunological second messengers by designer cells could be relevant in a panoply of diseases associated with immune dysregulation, which could benefit from closed-loop therapies. For example, autoimmune pathologies would be excellent targets. Graft cells can be modified to respond to pro-inflammatory cytokines in order to treat diseases affecting the cartilage in joints. By engineering stem cells to secrete an inactive soluble TNFα receptor deficient in signal transduction, a local reduction of inflammation was achieved upon their differentiation into cartilage and transplantation into joints as a treatment strategy for rheumatoid arthritis (Brunger et al., 2017) (Figure 3A). Inflammation is one of the main factors driving the progression of joint pathologies, so this approach is of particular interest for regenerative medicine.

Psoriasis is another chronic inflammatory condition, and is associated with elevated levels of both TNF and IL22. By using AND gate logic in designer cell implants to couple the signaling from both receptors that are activated by these cytokines, it was possible to inducibly trigger gene expression and release of the anti-inflammatory cytokines IL4 and IL10. This system prevented psoriatic flares and restored normal skin morphology in mice (Schukur et al., 2015) (Figure 3B).

In aging populations, high urate concentrations can lead to the development of gout, a painful form of arthritis caused by the deposition of urate crystals in joints (Dalbeth et al., 2016). A therapeutic landmark towards gout treatment was achieved by developing a designer cell implant capable of restoring the homeostasis of urate metabolism in rodents. An engineered *Deinococcus radiodurans*-derived uric acid sensor was used to control the expression of secreted *Aspergillus flavus* urate oxidase. This enzyme reduced uric acid concentration to sub-pathological levels and prevented crystal formation and kidney damage in urate oxidase-deficient mice (Kemmer et al., 2010) (Figure 3C).

Allergic pathologies are an increasing problem, especially in developed countries (Martinez and Holt, 1999). Such allergic responses are often associated with pathological levels of IL4 and IL13. Rerouting of IL4 and IL13 signaling into chimeric promoters for the production of an engineered DARPin that binds to and antagonizes human IgE reduced the release of histamine from IgE-stimulated degranulating basophiles, dampening the pro-inflammatory allergic response and preventing overactivation of the immune system in a closed-loop reversible manner (Chassin et al., 2017) (Figure 3D). Cytokines can also be used to direct the migration of certain types of engineered cells, e.g., immune cells, keratinocytes, stem cells or their engineered functional equivalents, and to improve their localization or enable the detection of specific phenotypes, such as senescence-associated secretory phenotypes (Coppé et al., 2010). The senescence cytokine IL6 has been used to trigger calcium release from the ER by an engineered IL6R-VEGFR2 receptor. This calcium second messenger then activates an engineered calcium-sensing RhoA variant, leading to actin remodeling that causes cells to migrate along an increasing IL6 concentration gradient (Qudrat et al., 2017) (Figure 3E). This could be used in the future development of highly targeted anti-cancer therapies, as it provides options for recruiting, activating or potentiating engineered immune cells at the precise location where their activity is required. Another notable example of immune system activation leading to a systemically detectable physiological response is fever. Through the engineering of a temperature-sensing transcription factor with a temperature optimum in the fever range it was possible to reversibly activate gene expression in mammalian cells with sub-degree precision. Gene expression from a promoter operated by this transcription factor was activated in a mouse model of pyrexia (Stefanov et al., 2021) (Figure 3F). The universal character of the developed fever sensor and its orthogonal promoter provides a basis for treating either the fever-causing underlying condition or the increased body temperature itself in a closed-loop therapeutic circuit.

### Closed-loop regulation by small molecules or peptide endocrine signals

**FIGURE 4:**
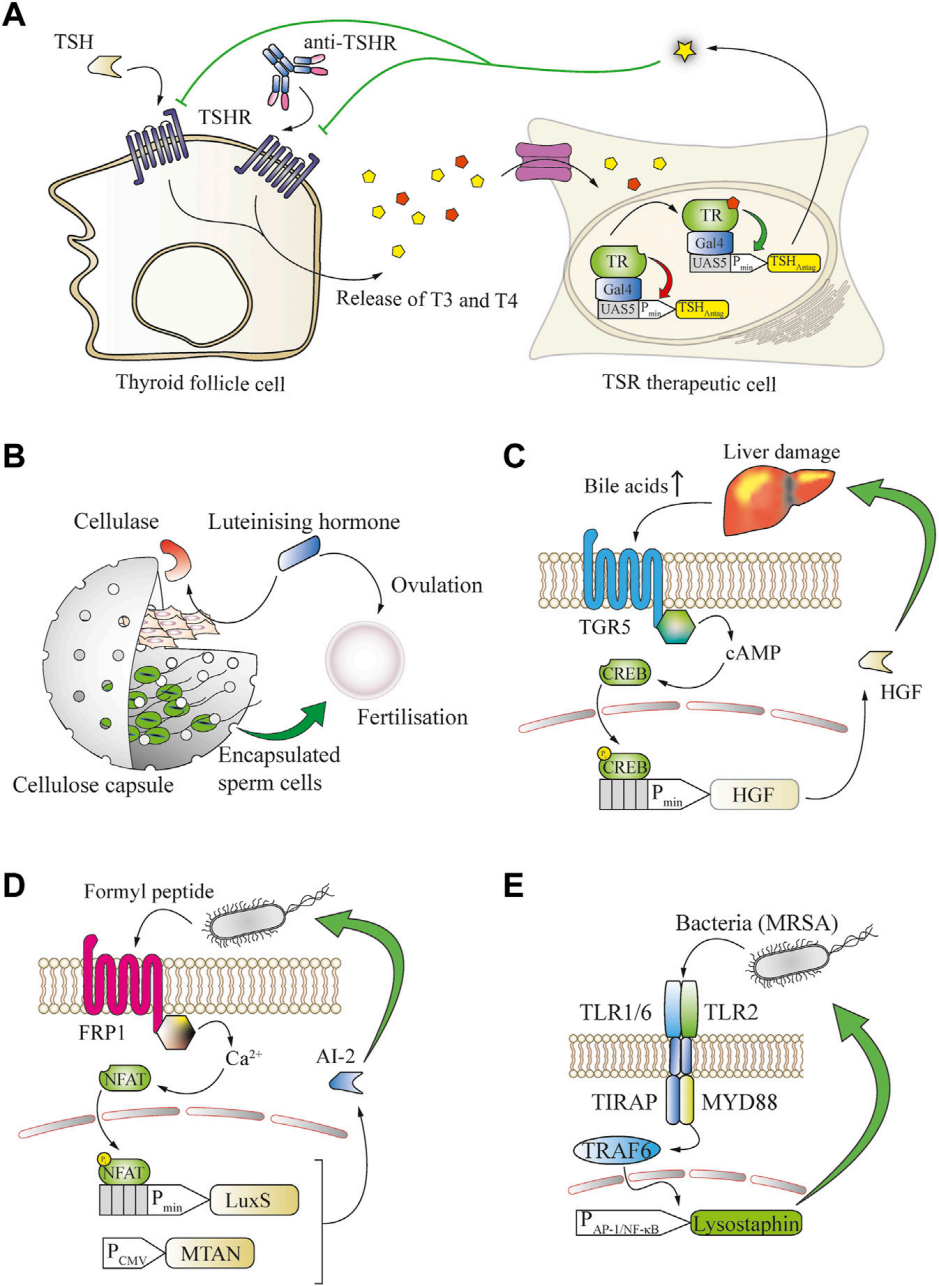
Many small molecules can be used as biomarkers to enable closed-loop therapeutic circuits in engineered mammalian cells. **(A)** Graves’ disease can be treated in a closed loop by T3/T4-responsive production of a thyroid-stimulating hormone receptor (TSHR) antagonist. Thyroid follicle cells release increased amounts of the thyroid T3 and T4 hormones upon overstimulation by TSH or antibodies targeting the TSH receptor. The released thyroid hormones bind to an engineered fusion protein of the thyroid receptor to a Gal4 DNA-binding domain and activate the expression of a TSH antagonist from a chimeric promoter, which potently blocks the overactivation of TSH receptors. **(B)** Bovine fertilization *in vivo* is enabled through closed-loop control of the *in utero* release of sperm cells by the ovulation-triggering luteinizing hormone. LH induces the expression of cellulase by co-encapsulated engineered cells, resulting in degradation of the capsule and release of the sperm cells. **(C)** Bile acid serum levels are an indicator of liver damage and can enable closed-loop hepatocyte protection. An increase in serum concentration of bile acids activates the TGR5 GPCR expressed by engineered cells, which converges into the intracellular cAMP cascade to trigger the expression of hepatocyte growth factor (HGF) from a CREB-operated promoter, limiting the liver damage. **(D)** N-Formyl peptide, a typical bacterial signature biomarker, enables closed-loop release of quorum-sensing interference molecules from engineered mammalian cells. Upon binding of the biomarker to the FRP1 GPCR, an intracellular calcium influx is triggered that activates the NFAT transcription factor and induces expression of the S-ribosylhomocysteine lyase LuxS, which, together with the constitutively expressed MTAN, enables the production of the *Pseudomonas aeruginosa* quorum-sensing signal molecule AI-2. **(E)** Bacterial cell-wall components can serve as biomarkers for engineered bactericidal cells. The presence of bacteria, including methicillin-resistant *S. aureus*, is sensed by heterodimeric receptors formed by TLR2 and the TLR1 or TLR6 chains. These receptors activate TRAF6 *via* MYD88 and enable inducible expression of the potent bactericidal peptide lysostaphin from a minimal AP-1 or NF-κB promoter, leading to elimination of the pathogens.

In many chronic diseases, natural feedback loops become imbalanced and changes of small molecules or peptides can be detected and employed as biomarkers. For example, in Graves’ disease, hyperthyroidism is caused by agonistic autoantibodies that trigger overactivation of the thyroid-stimulating hormone receptor (TSHR) (McIver and Morris, 1998). By rerouting T3-induced thyroid-sensing receptor (TR) signaling for the production of an antagonist of the thyroid-stimulating hormone receptor (TSHR), it was possible to restore HPA homeostasis in hyperthyroid mice (Saxena et al., 2016) (Figure 4A).

Closed-loop systems are also applicable for endocrine signaling, for example to promote bovine insemination. For this purpose, sperm cells were co-encapsulated in cellulose-based implants with designer cells that expressed cellulase upon detection of luteinizing hormone, which is a trigger of ovulation. The expressed cellulase degraded the cellulose capsules, enabling the release of sperm at the desired time-point during ovulation of the animals, and led to successful fertilization (Kemmer et al., 2011) (Figure 4B).

Increased blood bile-acid concentrations are often a biomarker for persistent liver damage. A metabolic GPCR receptor capable of sensing bile-acid concentration has been rerouted in therapeutic cells to produce a liver-protective protein that was able to prevent acute drug-induced liver failure in mice (Bai et al., 2016) (Figure 4C). Another major area where feedback-controlled therapeutics can provide significant clinical benefits is the ongoing fight against bacterial pathogens. Many bacterial communication mediators are small molecules, and various domains exist that can be engineered into mammalian cells to detect bacterial quorum-sensing signals. Such approaches could establish ways to interact with and control pathogenic infections through disruption of bacterial organization. For example, mammalian cells were equipped with the FRP1 receptor to detect formyl peptide sensor (FPS) and reroute its signaling for the expression of LuxS. In combination with 5′-methylthioadenosine/S-adenosylhomocysteine nucleosidase (MTAN) this enabled the production and release of the quorum-sensing interference molecule autoinducer 2 (AI-2) of *Vibrio harveyi*, which interfered with biofilm formation by the pathogenic yeast *Candida albicans* (Sedlmayer et al., 2018b) Figure 4D). Furthermore, to program the dysregulation of quorum sensing of *Pseudomona aeruginosa*, its autoinducer 1 (PAI-1) was detected by engineered mammalian cells expressing a *Pseudomonas* autoinducer sensor (PAiS). This enabled the release of autoinducer-inactivating enzyme MomL from a LasR-operated promoter, as well as the biofilm-degrading hydrolases PslG and PelA (Sedlmayer et al., 2017). These approaches are focused on the prevention of biofilm formation or enhancing the host immune function, but not pathogen killing, which might require additional treatment (Yu et al., 2003). In order to address this issue, mammalian cells were programmed to directly detect pathogenic bacteria by introducing a genetic circuit based on the toll-like receptors 2 and 1 or 6, and their signaling was rerouted to an NF-κB-operated chimeric promoter for the inducible production of the highly efficient bactericidal peptide lysostaphin. In a foreign-body infection model in mice, this system was able to resolve acute MRSA infection that was refractory to the gold standard vancomycin treatment (Liu et al., 2018a) (Figure 4E).

### Open-loop circuits that can enable behavior-based closed-loop operations

**FIGURE 5:**
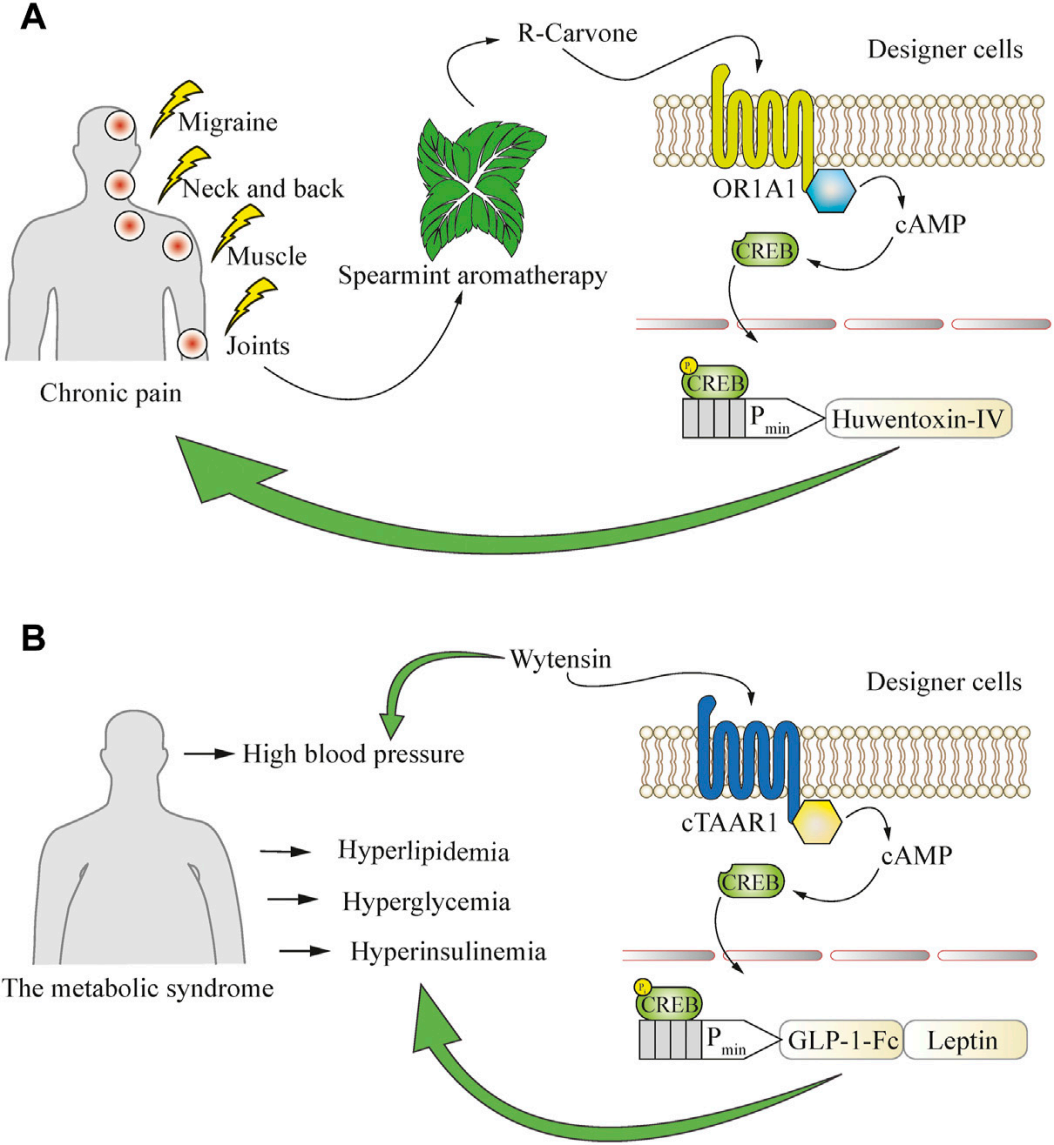
Using a defined behavior to convert open-loop cellular systems into closed-loop circuits. **(A)** Application of aromatherapy for the treatment of chronic pain can be used to drive closed-loop production of an analgesic peptide that blocks the pain-conductive Na_V_1.7 channels. Inhaled R-carvone, a volatile substance commonly found in spearmint aromatherapy, is used as a specific activator of the OR1A1 GPCR in engineered cells. This receptor potently induces the cAMP pathway, which is then rerouted to a CREB-operated promoter for the production of huwentoxin-IV to boost the therapeutic effect of aromatherapy. **(B)** Engineered cells enable closed-loop combinatorial treatment of metabolic syndrome co-morbidities such as hyperlipidemia, hyperglycemia, hyperinsulinemia, and hypertension, which are often simultaneously present in patients. Oral intake of the anti-hypertensive drug wytensin specifically activates cTAAR1 GPCR in engineered cells to enable the production of protein drugs addressing the co-morbidities. The cTAAR1 receptor potently induces the cAMP pathway, which is rerouted to a CREB-operated promoter for the production of engineered GLP-1 to treat hyperglycemia and hyperinsulinemia, and leptin to address hyperlipidemia.

Behavioral cues can also be used to generate designer cell responses. One such case is chronic pain, where capsaicin patches and spearmint aromatherapy are commonly applied as nearly side-effect-free remedies to ameliorate the symptoms. Cells responsive to the spearmint aromatic molecule R-carvone were engineered to produce the analgesic peptide huwentoxin-IV, which is a safe and potent Na_V_1.7 channel inhibitor, and resulted in a decrease of pain-related behavior in mice after inhalational aromatherapy (Wang et al., 2018). This system employed the R-carvone-responsive olfactory receptor OR1A1 and the olfactory-specific G protein (G_olf_) to achieve spearmint-inducible gene expression (Figure 5A). Such an approach could combine the beneficial effects of standard medications or medical procedures and cellular therapies, and also be used as a delivery system for polypeptide therapeutics that might be degraded if ingested directly. Such peptide-based therapeutics could target natural sensors and signaling pathways and act agonistically or antagonistically on various physiological processes (Carter and Lazar, 2018) and coupling to behavioral cues would provide new-to-nature behavioral homeostasis control.

Such combinatorial therapies could also expand the applicable range of medical conditions. Chronic diseases rarely come without comorbidities, and cell-based therapeutics can be programmed to respond to conventional treatment strategies directed towards particular aspects of the condition. For example, typical metabolic syndrome symptoms such as hyperlipidemia, hyperglycemia, and hyperinsulinemia may co-occur with hypertension (Katsimardou et al., 2020). This observation has been utilized to develop an experimental implant sensitive to the small-molecule drug wytensin used for treatment of hypertension (Ye et al., 2013). The intracellular signaling cascade triggered by wytensin through activation of the trace amine receptor cTAAR1 was rewired to induce GLP1 and leptin expression from synthetic CREB-operated promoters and enabled the treatment of metabolic syndrome-associated hyperlipidemia, hyperglycemia and hyperinsulinemia, which are often an underlying cause of progressive hypertension (Charlton, 2009) (Figure 5B). Although still at the proof-of-concept stage, such combinatorial approaches involving conventional medications together with engineered cells could soon provide a therapeutic advantage in the clinic, as hypertension is a common symptom and a significant co-morbidity risk factor, even though it is not the underlying disease.

### Outlook for neurological conditions

**FIGURE 6:**
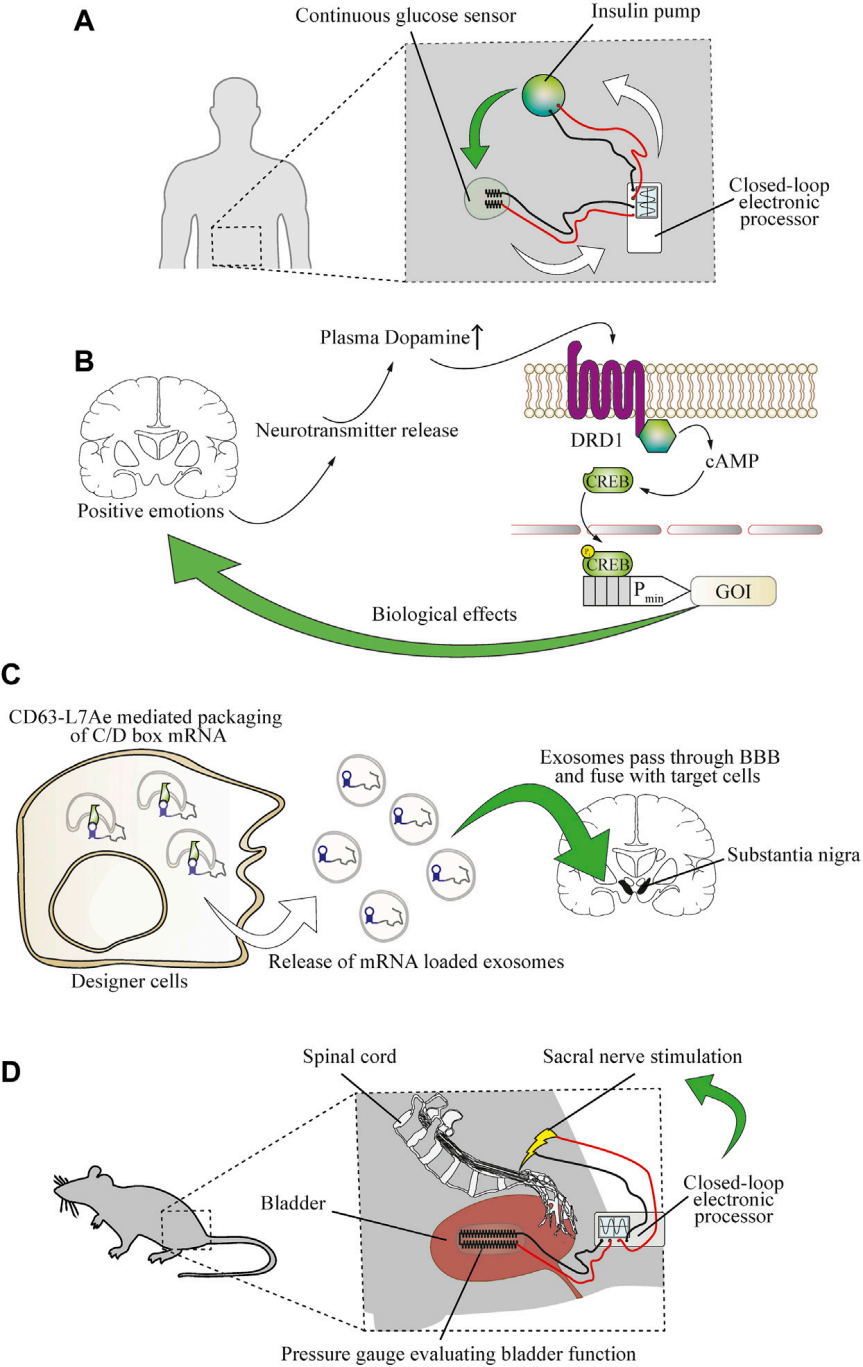
Establishing direct bidirectional electronics-to-physiology connections that would enable cellular therapies for neurological diseases. **(A)** Coupling of physiological parameters such as glucose concentration to electronic devices is possible and enables electronic closed-loop control of glycemia. A continuous glucose sensor evaluating glycemia sends information to a wearable electronic processing device that controls the function of an insulin pump. Upon release of insulin, glycemia is decreased, and the decrease is detected by the glucose sensor, causing the controller device to reduce the function of the insulin pump. Red and black lines indicate wiring. **(B)** The molecular patterns of specific types of brain activity can be detected by engineered cellular implants. Positive emotions, such as sexual arousal, were used to reroute neurotransmitter release in the brain to establish direct control over cellular implants in the periphery. The DRD1 GPCR is activated by increased blood dopamine levels and triggers cAMP signaling, resulting in phosphorylation of CREB and expression of a gene of interest (GOI) under its control. **(C)** Engineered cellular implants can deliver therapeutics directly to the brain. An exosome delivery strategy enables treatment of neuroinflammation by engineered therapeutic cells in a mouse model of Parkinson’s disease. The mRNA encoding for the therapeutic protein is tagged with a C/D box sequence that is recognized by a CD63-L7Ae fusion protein, enabling packaging into lipid bilayer vesicles. Upon release into the bloodstream, the exosomes pass the blood-brain barrier (BBB) and deliver the mRNA cargo to target cells in the brain. **(D)** Coupling of physiological functions to electronic devices enables closed-loop control over urinary incontinence. An implanted electronic gauge constantly measures the pressure in the bladder to evaluate its function. The signal is transferred to an electronic processor device that induces stimulation of the sacral nerve to prevent overemptying of the bladder.

Treatment of chronic disease requires prolonged or sometimes life-long delivery of therapeutics or treatment procedures. The function of biomedical devices can be enhanced through interfacing them with biomarkers in a closed-loop operational design, or their longevity could be extended by using self-replenishing engineered cells (Krawczyk et al., 2020b). A prominent example of this is provided by insulin pumps that can be operated in response to the input signal from a glucose sensor in an electronic hybrid-type closed-loop model (Leelarathna et al., 2021) (Figure 6A). As digital technology advances, it seems likely that autonomous cell-based therapeutics will increasingly shape the future of personalized medicine (Sedlmayer et al., 2018a). In the case of the nervous system, biomarkers can include changes in the electrical activity of the brain (Iosifescu, 2011). By focusing on small-molecule neurotransmitters, cellular therapeutics were developed to sense the molecular pattern of happiness-associated feelings and to treat hypertension based on this. Designer cells were constructed to reroute the dopamine receptor D1 (DRD1)-triggered increase of intracellular cAMP to a CREB-controlled minimal promoter for expression and release of atrial natriuretic peptide, and an implant containing these cells restored blood pressure to pre-hypertensive levels (Rössger et al., 2013a) (Figure 6B). Coupling a pathological neural activity to engineered cells could enable closed-loop treatment options for neurological diseases. However, cell implant-based treatments for most central nervous system diseases remain to be established. Among the main reasons for this is the blood-brain barrier, which prevents the entry of most peptide-based drugs into the brain. However, advanced delivery techniques, such as cell-generated extracellular vesicles and exosomes, have enabled the efficient delivery of mRNA into the brain in mice, and ameliorated neurotoxicity and neuroinflammatory damage in a mouse model of Parkinson’s disease (Kojima et al., 2018) (Figure 6C). Alternatively, cell therapies could be electrically coupled (Krawczyk et al., 2020b) to mechanical devices either as biomarker sensors or for the treatment of neurological disorders where neuromodulation is required. However, such mechanical implants face major challenges. They should be non-immunogenic, should not trigger coagulation, should be resistant to bacterial biofilm formation, and should be durable and remain operational for long periods (Bose et al., 2020). In contrast, maintenance is not an issue in purely cellular implants, because of the natural cellular repair and replenishment processes. Nonetheless, biomedical devices interfacing with the electrical activity of the brain have shown promising pre-clinical results. Vagal nerve stimulation in response to cholecystokinin (CCK) or distention of the stomach was used as a neuron-based closed-loop treatment for obesity (Cork et al., 2018). Further, urinary incontinence in a rat model was ameliorated by sacral nerve stimulation based on a soft strain gauge directly evaluating bladder function (Mickle et al., 2019) (Figure 6D). In addition, real-time electroencephalogram (EEG) measurements of the slow oscillation rhythm of the brain were used for closed-loop auditory in-phase stimulation during sleep to improve declarative memory in humans (Ngo et al., 2013). Closed-loop neuromodulation based on biomarkers of pathological activity (Paz et al., 2013) or mitochondrial function (Adams et al., 2018) could offer substantial clinical benefits in comparison to open-loop deep-brain stimulation in Parkinson’s and seizure patients. Integrating electrical neurological devices in a closed-loop with designer cell implants is currently a challenging task, but has the potential to overcome the drawbacks of cellular and mechanical therapies and to enable the treatment of highly complex neurological diseases in the future.

## Concluding remarks

Cellular closed-loop therapeutic devices have the potential to treat a range of hard-to-manage diseases. Additionally, they could improve the quality of life and the therapeutic compliance of patients, since they are readily adaptable to personalized needs and life-style. However, despite recent advances towards more intelligent and autonomous delivery of medication, extensive clinical research will still be needed to validate closed-loop therapeutics before their introduction into clinical practice, especially to ensure the safety and longevity of engineered cellular-based prosthetic implants. These key parameters depend upon many factors, including the selected cell type, whether allo- or syngeneic cells are being used and whether interaction with the host cells is desirable, as in the case of CAR-T therapies, or whether encapsulation to protect the engineered cells from the host’s immune system would be preferable. In the case of the second option, further research would also be necessary to evaluate different implantation techniques. For example, it may be possible to utilize immune-privileged areas of the body to localize and contain the prosthetic implants, or otherwise it would be necessary to further improve the materials used to establish an immunoprotected encapsulating environment for the implanted cells. Materials used for encapsulation should permit an adequate nutrient flux to the implant to ensure its proper function and longevity, while minimizing detectability by the immune system and antigen shedding. If syngeneic or immunologically compatible patient-derived cells are to be used, it would be necessary to optimize the *ex-vivo* modification processing and expansion of these cells to generate successful allografts. These improvements may include both the gene modification and stable integration strategies, where it is critical to avoid or detect potentially carcinogenic integrations, as well as methods to screen the best-performing engineered cells based on defined criteria to obtain the most efficient allografts. The selected designer cells should be robust to prevent frequent replacements and growth-attenuated to avoid any overpopulation of the implant devices. Engineering of patient-derived generalized stem cell populations or the usage of iPSC, followed by expansion and subsequent differentiation towards a terminally differentiated cell fate before implantation, may prove to be another approach for transferring cellular closed-loop therapies to the clinic. This approach has the added benefit that stem cells could be collected before the development of the disease and stored frozen for long periods until required, since many pathophysiological conditions negatively affect the condition and viability of the patient’s own cells and thus may render *ex-vivo* engineering more inefficient or even unsuccessful.

Another current focus of research in gene therapy is direct *in vivo* engineering of the patient’s cells, which could be highly beneficial in the case of genetic diseases caused by point mutations, which might be cured by the use of engineered base editors. Further advances in current delivery methods, such as adenoviral (AV) or adeno-associated viral (AAVs) particles for the delivery of larger DNA fragments, might also make it possible to deliver longer gene fragments including those encoding complete closed-loop therapeutic circuits. It should be noted that modern viral vector-based strategies focus on establishing episomes, which are extrachromosomal DNA particles, in order to reduce the risk of integration into tumor suppressor genes.

Furthermore, there is still room for improvement of the closed-loop circuitry itself, as not all disease biomarkers have natural receptors that could be used to trigger the expression of therapeutic proteins from engineered cells. Prominent examples would be biomarkers of a physical nature, such as neuronal electrical discharges and increase in body temperature (fever), or intracellular proteins that are systemically released due to necroptosis of a specific cell type. As we have described, synthetic biology enables new-to-nature receptors to be constructed through rational design and the combination of different functional modules, thus enabling the detection of such biomarkers. Expanding the range of detectable cues would further extend the scope of closed-loop systems, as multiplexing of several disease biomarkers would be possible and should increase the therapeutic specificity. Finally, to enable the treatment of conditions that require an immediate response, such as anaphylactic shock, closed-loop circuits could be integrated into specialized endocrine cells that contain intracellular protein storage vesicles (Krawczyk et al., 2020b). Upon activation of these cells their granules are fused within minutes to the cell membrane, releasing large quantities of therapeutic cargo that can immediately counteract the pathological condition.
